# Social media use and depression among middle-aged and older Chinese adults: mediating roles of health literacy and perceived social support

**DOI:** 10.3389/fpubh.2026.1817281

**Published:** 2026-05-04

**Authors:** Yihua Liu, Xiaoge Zhao

**Affiliations:** 1 Department of Sociology and Psychology, School of Public Administration, Sichuan University, Chengdu, China; 2 College of International Tourism and Public Administration, Hainan University, Haikou, China

**Keywords:** depression, health literacy, middle-aged and older adults, perceived social support, social media use

## Abstract

**Background:**

Most research on social media use and depression has focused on young adults. A few studies in middle-aged and older Chinese adults have reported a negative association, but the underlying mechanisms remain unclear.

**Methods:**

A cross-sectional survey was conducted among middle-aged and older adults (*n* = 2,743) in five cities of Hainan Province, China. Participants completed the Patient Health Questionnaire-9, the Social Media Use Integration Scale, the Short-Form Health Literacy Questionnaire, and the Perceived Social Support Scale. Mediation and moderation models were tested using the PROCESS macro with bootstrap methods.

**Results:**

Social media use was significantly negatively associated with depression (*B* = −0.045, *p* < 0.001). Health literacy (*B* = −0.009, 95% *CI* [−0.013, −0.005]) and perceived social support (*B* = −0.015, 95% *CI* [−0.019, −0.011]) mediated this relationship. Education level strengthened the positive associations of social media use with health literacy (*B* = 0.003, *p* < 0.05) and perceived social support (*B* = 0.019, *p* < 0.001).

**Conclusion:**

Social media use was associated with lower levels of depression among middle-aged and older adults. Health literacy and perceived social support may serve as potential pathways underlying this association. In addition, these associations varied by education level.

## 1 Introduction

Depression is a major public health concern and shows a high prevalence among middle-aged and older adults (1, 2). An epidemiological survey reported that the lifetime prevalence of depression among Chinese adults aged 50 years and older exceeds 7%, which is significantly higher than that in other age groups (3). Depression not only impairs emotional wellbeing but is also closely linked to cognitive decline. Previous studies have shown that depression accelerates memory deterioration and impairs executive function, thereby reducing individuals' capacity for daily functioning (4). Moreover, depression frequently co-occurs with chronic physical conditions, such as cancer and cardiovascular diseases (5, 6). This comorbidity significantly increases disease burden and mortality risk. China is undergoing rapid population aging, and the size of the middle-aged and older population continues to expand. Mental health problems in this group have therefore become an important public health issue.

With the rapid development of digital technology, social media has become embedded in the daily lives of middle-aged and older adults and has emerged as a key tool for information acquisition and social interaction. Official statistics indicate that by June 2025, the number of social networking users in China had reached 1.107 billion, with a steadily increasing proportion of middle-aged and older users (7). Social media platforms such as WeChat and TikTok are frequently used in their daily lives. In this context, social media use plays an increasingly important role in influencing the psychological wellbeing of middle-aged and older adults. However, research on the association between social media use and depression has disproportionately focused on young people (8, 9). Social media has often been framed as a risk factor, with emphasis on addiction, information overload, and social comparison (10–12). In contrast, middle-aged and older adults have long remained at the margins of this literature. Although several studies based on Chinese samples have reported a negative association between social media use and depression in this population (2, 13), the underlying mechanisms remain unclear. This gap limits understanding of the potential positive effects of social media and constrains the development of targeted interventions for middle-aged and older adults.

Social cognitive theory provides an important framework for understanding the development of depression among middle-aged and older adults (14). The theory emphasizes continuous reciprocal interactions among behavior, personal factors, and environmental influences (15). Individuals are not passive recipients of external stimuli. Instead, they actively process information and engage in social interactions to acquire cognitive and social resources, which further influence their mental health. In the digital era, social media use has become a key daily behavior among middle-aged and older adults, affecting their information acquisition and social interactions. Based on social cognitive theory, this study examines the mechanisms linking social media use to depression among middle-aged and older adults in China. The findings aim to provide empirical evidence for developing targeted mental health promotion strategies for this population.

### 1.1 Social media use and depression

Prior research on social media use and mental health has largely focused on young people and has primarily emphasized its potential risks. Considerable evidence indicates that high-frequency use and excessive dependence on social media are significantly associated with adverse psychological outcomes, including depression, anxiety, and low self-esteem (16–18). These negative effects operate through multiple pathways, such as intensified social comparison, exposure to cyberbullying, and reduced offline social interaction (19, 20). Young users are often motivated by the pursuit of social approval and peer acceptance (21). In the context of an unstable self-concept and strong self-esteem needs, immediate online feedback is more likely to generate psychological strain and amplify negative affect. Moreover, limited self-regulatory capacity among young individuals increases the likelihood of addictive or uncontrolled use, leading to cumulative psychological burden with greater exposure (22). In contrast, research on middle-aged and older adults remains relatively scarce, and available evidence suggests that their motivations of social media use and associated psychological outcomes differ from those of younger users (23). According to psychosocial development theory, individuals have different developmental tasks and needs across life stages (24). Young adults focus on identity formation and strive to clarify their values and life goals (23). They also face multiple pressures related to education, partner selection, and employment. Therefore, they are more likely to use media to expand social relationships and for self-presentation (25). In contrast, middle-aged and older adults enter a stage of reflecting on and evaluating life experiences (23). They also face multiple challenges, including work and family responsibilities and changes in health status. In this context, their media use motivations shift from relationship expansion to more instrumental purposes. Specifically, middle-aged and older adults primarily use social media to maintain existing social connections, obtain daily-life information, and engage in leisure activities (26, 27), rather than for social comparison or identity display. This more instrumental and relationship-oriented use may reduce loneliness, enhance perceived social support, and improve executive functioning (28, 29), thereby lowering the risk of depression. In addition, middle-aged and older adults tend to possess a more stable self-concept and more mature self-regulatory capacities, which may mitigate emotional fluctuations induced by social comparison and evaluative feedback. Evidence from diverse cultural contexts further indicates that social media use may contribute to positive psychological outcomes in later life, including the maintenance of cognitive functioning, enhanced self-efficacy, and greater life satisfaction (30–32). Social cognitive theory highlights reciprocal interactions among behavior, personal factors, and environmental influences (15). As an agentic behavior, social media use may influence emotional states by shaping cognitive processing of social information and restructuring social interaction contexts. In China, social media serves as a major channel through which middle-aged and older adults access public information, maintain family ties, and participate in community life. This pattern of use is characterized by strong instrumentality and relational embeddedness (26, 27), which is more likely to activate the positive cognitive appraisals and environmental support processes emphasized by social cognitive theory (33). For example, middle-aged and older adults may form positive outcome expectations through exposure to health information and obtain social support via family and community interactions. These processes may help alleviate their negative emotions and lower the risk of depression. Therefore, the following hypothesis is proposed:

**Hypothesis 1**. Social media use is negatively associated with depression among middle-aged and older adults in China.

### 1.2 Health literacy and perceived social support as mediators

Social cognitive theory proposes that media influence individuals through different pathways (34). The direct pathway emphasizes that media shape cognitive processing and behavioral choices by providing informational cues and behavioral models. As an important channel of information dissemination, social media provides middle-aged and older adults with access to health knowledge and opportunities for social learning (35). By exposure to health-related content and observation of others' behaviors, individuals can adjust their cognitive orientations and behavioral patterns. On the one hand, social media platforms aggregate extensive information on disease prevention, health management, and lifestyle practices (36, 37). Middle-aged and older adults can acquire health knowledge through repeated exposure and active searching. On the other hand, by observing others' shared health experiences and recovery stories online, individuals may develop positive outcome expectations for health behaviors through modeling and social reinforcement (38). This process facilitates the internalization of health-related knowledge and skills. These processes suggest that social media use may enhance health literacy among middle-aged and older adults. Furthermore, as a key cognitive resource, health literacy may play an important role in mental health (39). Studies show that higher health literacy enables individuals to better understand health risk information, form appropriate health beliefs, and adopt effective coping strategies, which may reduce psychological distress (40). A meta-analysis reported a significant negative association between health literacy and depression (41). Compared with individuals with lower health literacy, those with higher health literacy had approximately a 10% lower risk of depression. Similarly, a cross-sectional study found that higher health literacy was associated with a lower prevalence of generalized anxiety disorder (42). According to the health belief model, cognitive appraisals of disease susceptibility, severity, and coping efficacy shape emotional responses and health-related behaviors (43). Health literacy provides the foundation for these appraisals. Middle-aged and older adults with higher health literacy are more likely to develop a greater sense of control over their health, experience reduced concerns about illness and aging, and thus potentially alleviate depression (44). Taken together, health literacy may serve as a mediating mechanism linking social media use to depression. Therefore, the following hypotheses are proposed:

**Hypothesis 2**. Health literacy mediates the relationship between social media use and depression.
**Hypothesis 2a**. Social media use is positively associated with health literacy.
**Hypothesis 2b**. Health literacy is negatively associated with depression.

Social cognitive theory also posits that media can influence individuals through an indirect pathway (34). In this pathway, media connect individuals to social networks, which in turn may affect their cognition and behavior. Social media extends middle-aged and older adults' social interactions from offline settings to online spaces (28), facilitating the maintenance of ties with family members and friends. Through sustained emotional expression and information sharing, individuals receive responses and support from others and develop stable patterns of social feedback and reinforcement. These interaction processes enhance subjective feelings of being cared for, thereby increasing perceived social support (45). Empirical evidence shows a positive association between social media use and perceived social support among middle-aged and older adults, mainly because social media promotes social connection, reduces social isolation, and increases opportunities for interaction (28, 35). Middle-aged and older adults often use social media to maintain social relationships, which facilitates the conversion of online interactions into perceivable support resources. Furthermore, perceived social support is widely regarded as a key protective factor for mental health (46). According to the stress-buffering model, social support mitigates the adverse psychological effects of stressful events by providing emotional comfort, informational guidance, and practical assistance, thereby reducing the likelihood of depression (47). Many empirical studies also indicate that higher perceived social support helps alleviate loneliness, reduce the risk of depression, and enhance quality of life, especially when facing health challenges (48, 49). For example, Grey et al. (50) surveyed over 2,000 adults during the COVID-19 pandemic and found that those with higher perceived social support were 63% less likely to report depressive symptoms than those with lower perceived social support. For middle-aged and older adults, reliance on social support increases as social roles change and physical functioning declines. Perceived social support therefore plays a particularly important role in their mental health. Taken together, perceived social support may serve as another mediating mechanism linking social media use to depression. Therefore, the following hypotheses are proposed:

**Hypothesis 3**. Perceived social support mediates the relationship between social media use and depression.
**Hypothesis 3a**. Social media use is positively associated with perceived social support.
**Hypothesis 3b**. Perceived social support is negatively associated with depression.

### 1.3 Education level as a moderator

Individual cognitive attributes influence how individuals actively process environmental information and transform observed or enacted behaviors into cognitive and social resources (15). Education level is an important indicator of cognitive capacity, information-processing skills, and learning experience. In digital contexts, the health information and social interaction opportunities provided by social media do not exert uniform effects across individuals (51). Their impact largely depends on individuals' ability to comprehend, evaluate, and internalize information. Individuals with higher education levels generally possess stronger reading comprehension, information discrimination skills, and critical thinking abilities (52). They are therefore more likely to extract valuable health information from social media and translate it into actionable knowledge and behavioral strategies, thereby strengthening the positive association between social media use and health literacy. In contrast, individuals with lower education levels may experience greater cognitive load in information filtering and meaning construction, making it more difficult to convert online information into stable cognitive resources. At the same time, education level may also moderate the association between social media use and perceived social support. On the one hand, higher education is often associated with greater social capital and more advanced communication skills, which facilitate the formation and maintenance of supportive relationships in online interactions (53). On the other hand, individuals with higher education levels tend to report higher self-efficacy (54). During social interactions, they are more likely to interpret supportive cues positively and thus more effectively transform online communication into social support resources. By contrast, although individuals with lower education levels also engage in online communication, their interactions are more embedded in existing kinship or acquaintance networks, and the types and sources of support they receive are relatively limited. This constrains the positive relationship between social media use and perceived social support. Taken together, education level may serve as a boundary condition for the relationships between social media use and both health literacy and perceived social support. Therefore, the following hypotheses are proposed:

**Hypothesis 4**. Education level strengthens the positive association between social media use and health literacy.
**Hypothesis 5**. Education level strengthens the positive association between social media use and perceived social support.

### 1.4 Present study

**Figure 1:**
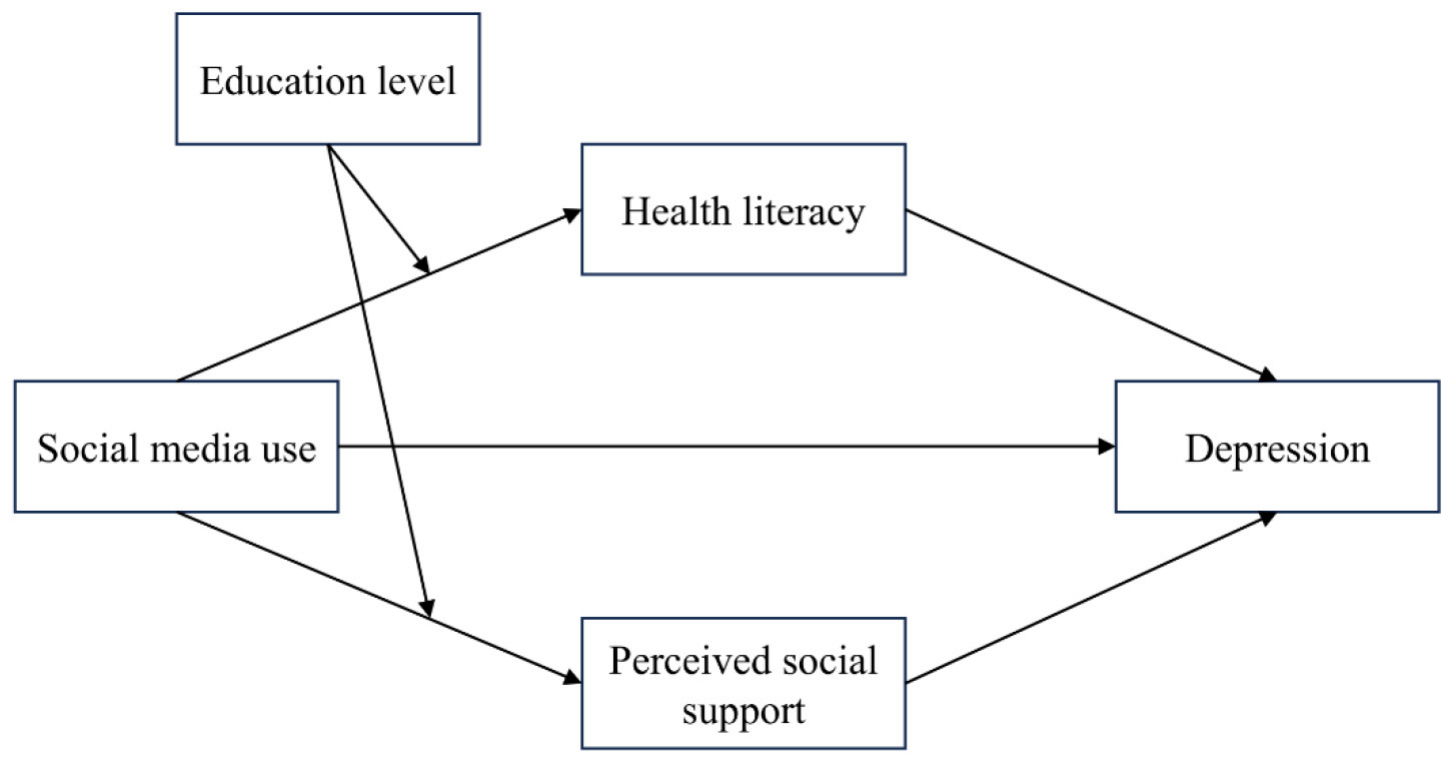
Moderated mediation model of the present study.

Previous research on the association between social media use and depression has focused primarily on young populations (8, 9). The present study shifts attention to middle-aged and older adults. Grounded in social cognitive theory, it develops a moderated mediation model (see Figure 1). The model examines how social media use, as a behavioral factor, may be linked to depression through cognitive resources such as health literacy and social resources such as perceived social support. In addition, education level may moderate how effectively individuals leverage social media use to acquire cognitive and social resources, thereby shaping its indirect effect on depression.

## 2 Methods

### 2.1 Participants and procedures

Consistent with prior research, individuals aged 45–59 years were defined as middle-aged, and those aged 60 years and above were defined as older adults (2, 13). The study was conducted in Hainan Province, China, from June to September 2025. In this province, more than 17% of the population is aged 60 years or older (55), and it is one of the Chinese provinces with the highest life expectancy (56), making it a suitable setting for research on the aging population. A multistage stratified cluster sampling method was employed. First, Hainan Province was stratified by geographic region (eastern, western, southern, northern, and central), and one city was randomly selected from each stratum. Within each selected city, several communities were randomly chosen, resulting in a total of 18 communities. Eligible participants were social media users (e.g., WeChat, TikTok) aged 45 years or older and capable of completing the questionnaire. Community workers assisted with participant recruitment, and trained investigators distributed and collected the questionnaires. A total of 2,977 questionnaires were distributed, and 2,804 were returned, yielding a response rate of 94.19%. After data cleaning and quality control, questionnaires with missing key information or inconsistent responses were excluded, resulting in a final sample of 2,743 participants. All participants provided written informed consent before participation. The study strictly followed the principles of the Declaration of Helsinki and was approved by the Ethics Committee of Hainan University (Approval No. HNASU-2025-20).

Participants' sociodemographic characteristics are presented in Table 1. Specifically, 62.60% were aged 45–59 years, and 37.40% were aged 60 years or older. Males accounted for 51.77% of the sample, and females for 48.23%. Regarding marital status, 88.33% of participants were married, while 3.46% were never married, 2.52% divorced, and 5.69% widowed. In terms of income, 30.59% reported a monthly per capita household income of CNY 3,000 or less, 39.85% reported between CNY 3,001 and CNY 6,000, 16.22% reported between CNY 6,001 and CNY 9,000, and 13.34% reported CNY 9,001 or above. In addition, 70.18% resided in urban areas, and 29.82% in rural areas.

**Table 1:** Sociodemographic characteristics of participants.

Characteristics	Number of participants (*n*)	Percentage (*%*)
Age		
45–59	1,717	62.60
≥60	1,026	37.40
Gender		
Male	1,420	51.77
Female	1,323	48.23
Marital status		
Married	2,423	88.33
Never married	95	3.46
Divorced	69	2.52
Widowed	156	5.69
Family per capita monthly income		
≤ CNY 3,000	839	30.59
CNY 3,001–CNY 6,000	1,093	39.85
CNY 6,001–CNY 9,000	445	16.22
≥CNY 9,001	366	13.34
Residence		
Urban	1,925	70.18
Rural	818	29.82

### 2.2 Measures

#### 2.2.1 Depression

The Patient Health Questionnaire-9 was used to assess participants' levels of depression (57). This scale has been widely applied in Chinese populations and has demonstrated good reliability and validity (58, 59). It consists of nine items (e.g., “Over the last 2 weeks, having thoughts that you would be better off dead or of hurting yourself in some way”). Each item is rated on a 4-point scale from 0 (not at all) to 3 (nearly every day), yielding a total score ranging from 0 to 27. Higher scores indicate higher levels of depression. The Cronbach's α of this scale in this study was 0.928.

#### 2.2.2 Social media use

The Social Media Use Integration Scale was used to measure social media use (60). Although originally developed for Facebook, Jenkins-Guarnieri et al. (60) suggested that the scale possesses adequate flexibility for use across different online social media platforms. Accordingly, the term “Facebook” in the original scale was replaced with “WeChat/TikTok” in the present study to enhance its contextual relevance in China. The scale has demonstrated good reliability in Chinese populations (61). It consists of 10 items (e.g., “I enjoy checking my WeChat/TikTok account”) rated on a 6-point Likert scale from 1 (strongly disagree) to 6 (strongly agree). Total scores range from 10 to 60, with higher scores indicating greater social media engagement. The Cronbach's α of this scale in this study was 0.837.

#### 2.2.3 Health literacy

The Short-Form Health Literacy Questionnaire was used to assess participants' levels of health literacy (62). This scale captures not only health literacy related to physical health but also individuals' ability to access and understand information for coping with mental health problems. It has demonstrated good reliability among middle-aged and older Chinese adults (35). The scale consists of 12 items (e.g., “Finding information on how to manage mental health problems such as stress or depression”) rated on a 4-point Likert scale (1 = very difficult, 4 = very easy). Total scores range from 12 to 48, with higher scores indicating higher health literacy. The Cronbach's α of this scale in this study was 0.934.

#### 2.2.4 Perceived social support

The Perceived Social Support Scale was used to assess participants' levels of perceived social support (63). This scale has demonstrated good reliability in Chinese middle-aged and older populations (64). It consists of 12 items (e.g., “There is a special person who is around when I am in need”) rated on a 7-point Likert scale (1 = strongly disagree, 7 = strongly agree). Total scores range from 12 to 84, with higher scores indicating higher perceived social support. The Cronbach's α of this scale in this study was 0.954.

#### 2.2.5 Education level

Education level was measured in years of formal schooling. Higher values indicate higher levels of educational attainment.

### 2.3 Statistical analysis

Data analyses were conducted using IBM SPSS (version 22.0) and the PROCESS macro (65). First, Pearson correlation analyses were performed to examine the relationships among key variables. Next, the PROCESS macro was used to test mediation and moderation effects. Specifically, Model 4 was employed to assess the mediating roles of health literacy and perceived social support. A 95% confidence interval (CI) based on 5,000 bootstrap samples was generated, and a mediation effect was considered significant if the CI did not include zero (66). Model 7 was used to test the moderating effect of education level. Sociodemographic variables (age, gender, marital status, income, and residential area) were controlled as covariates in both mediation and moderation analyses. Prior studies have demonstrated their associations with depression among middle-aged and older Chinese adults (64), and previous research on social media use and depression in this population has also adjusted for these variables (2, 13). *p* < 0.05 was considered statistically significant.

## 3 Results

### 3.1 Preliminary analysis

As shown in Table 2, the mean scores (and standard deviations) of depression, social media use, health literacy, perceived social support, and education level were 5.31 (*SD* = 5.07), 41.79 (*SD* = 8.27), 35.56 (*SD* = 6.03), 60.63 (*SD* = 12.45), and 11.03 (*SD* = 4.86), respectively. Skewness and kurtosis values of all variables were within acceptable ranges, indicating that the normality assumption was not violated (67). Correlation analyses indicated that depression was significantly negatively correlated with social media use (*r* = −0.117, *p* < 0.001), health literacy (*r* = −0.130, *p* < 0.001), and perceived social support (*r* = −0.219, *p* < 0.001). Social media use was significantly positively correlated with health literacy (*r* = 0.379, *p* < 0.001) and perceived social support (*r* = 0.212, *p* < 0.001). In addition, education level was significantly positively correlated with social media use (*r* = 0.403, *p* < 0.001), health literacy (*r* = 0.337, *p* < 0.001), and perceived social support (*r* = 0.088, *p* < 0.001).

**Table 2:** Correlation analysis of key variables.

Variables	*M*	*SD*	Skewness	Kurtosis	1	2	3	4	5
1. Depression	5.31	5.07	1.19	1.56	1	–	–	–	–
2. SMU	41.79	8.27	−1.20	0.06	−0.117^***^	1	–	–	–
3. HL	35.56	6.03	−0.11	1.24	−0.130^***^	0.379^***^	1	–	–
4. PSS	60.63	12.45	−0.31	0.58	−0.219^***^	0.212^***^	0.401^***^	1	–
5. Education level	11.03	4.86	−0.62	−0.07	−0.020	0.403^***^	0.337^***^	0.088^***^	1

^***^p < 0.001. SMU, Social media use; HL, Health literacy; PSS, Perceived social support.

### 3.2 Mediation analysis

This study tested the mediating roles of health literacy and perceived social support in the relationship between social media use and depression. The results are presented in Table 3. First, the total effect of social media use on depression was significant (*B* = −0.045, *p* < 0.001), supporting Hypothesis 1. Further analyses indicated that social media use was positively associated with health literacy (*B* = 0.109, *p* < 0.001), and health literacy was negatively associated with depression (*B* = −0.083, *p* < 0.001). Hypotheses 2a and 2b were supported. Bootstrap analyses showed a significant indirect effect of social media use on depression through health literacy (*B* = −0.009, 95% *CI* [−0.013, −0.005]), supporting Hypothesis 2. Additionally, social media use was also positively associated with perceived social support (*B* = 0.202, *p* < 0.001), and perceived social support was negatively associated with depression (*B* = −0.073, *p* < 0.001). Hypotheses 3a and 3b were supported. Bootstrap analyses indicated a significant indirect effect of social media use on depression through perceived social support (*B* = −0.015, 95% *CI* [−0.019, −0.011]), supporting Hypothesis 3.

**Table 3:** Mediation analysis.

Effect type	Path	*B*	*SE*	*t*	*p*	*LLCI*	*ULCI*
Total effect	SMU → Depression	−0.045	0.006	−7.543	< 0.001	−0.056	−0.033
Indirect effect 1	SMU → HL	0.109	0.007	16.791	< 0.001	0.096	0.122
	HL → Depression	−0.083	0.016	−5.238	< 0.001	−0.115	−0.052
	SMU → HL → Depression	−0.009	0.002	–	–	−0.013	−0.005
Indirect effect 2	SMU → PSS	0.202	0.014	14.206	< 0.001	0.174	0.230
	PSS → Depression	−0.073	0.007	−10.124	< 0.001	−0.087	−0.059
	SMU → PSS → Depression	−0.015	0.002	–	–	−0.019	−0.011

SMU, social media use; HL, health literacy; PSS, perceived social support; SE, standard error; LLCI, lower limit 95% confidence interval; ULCI, upper limit 95% confidence interval.

### 3.3 Moderation analysis

**Figure 2:**
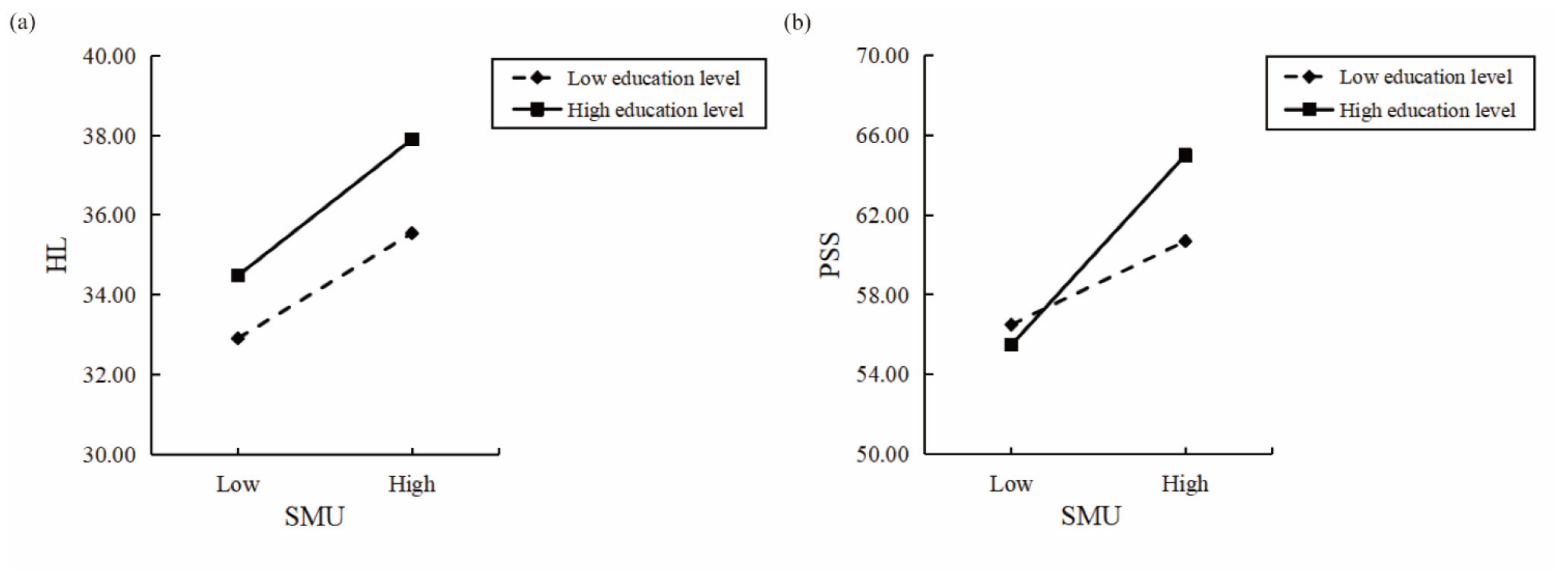
Simple slope analysis. SMU, social media use; HL, health literacy; PSS, perceived social support. **(a)** Moderating role of education level between SMU and HL. **(b)** Moderating role of education level between SMU and PSS.

This study tested the moderating role of education level in the relationship between social media use and health literacy. The results are presented in Table 4. When health literacy was the dependent variable, the interaction term between social media use and education level was significant (*B* = 0.003, *p* < 0.05), indicating a moderating effect. To further interpret this effect, simple slope analyses were conducted by dividing the sample into low and high education level groups (±1 *SD* from the mean). As shown in Figure 2, the positive association between social media use and health literacy was weaker in the low education level group (−1 *SD, B* = 0.091, *p* < 0.001) and stronger in the high education level group (+1 *SD, B* = 0.118, *p* < 0.001). These results indicate that higher education level strengthened the positive association between social media use and health literacy, supporting Hypothesis 4.

**Table 4:** Moderated mediation analysis.

Variables	HL				PSS			
	*B*	*SE*	*T*	*p*	*B*	*SE*	*t*	*p*
SMU	0.105	0.007	14.930	< 0.001	0.236	0.015	15.430	< 0.001
Education level	0.209	0.026	8.207	< 0.001	0.217	0.056	3.893	< 0.001
SMU × Education level	0.003	0.001	2.547	< 0.05	0.019	0.002	7.930	< 0.001
Age	0.010	0.057	0.178	0.859	1.009	0.125	8.067	< 0.001
Gender (ref = male)	−0.081	0.192	−0.423	0.672	1.327	0.420	3.159	< 0.01
Marital status (ref = married)								
Never married	−1.464	0.522	−2.807	< 0.01	−7.273	1.142	−6.372	< 0.001
Divorced	−1.186	0.602	−1.971	< 0.05	−5.198	1.317	−3.947	< 0.001
Widowed	−0.904	0.438	−2.065	< 0.05	0.607	0.958	0.634	0.526
Income	0.244	0.044	5.534	< 0.001	0.239	0.096	2.484	< 0.05
Residence (ref = Urban)	−0.761	0.231	−3.297	< 0.01	0.609	0.505	1.205	0.228
*R* ^2^	0.203	–	–	–	0.105	–	–	–
*F*	82.459	–	–	–	38.147	–	–	–

SMU, Social media use; HL, health literacy; PSS, perceived social support; SE, standard error.

In addition, this study examined the moderating role of education level in the relationship between social media use and perceived social support. The results are also presented in Table 4. When perceived social support was the dependent variable, the interaction term between social media use and education level was significant (*B* = 0.019, *p* < 0.001), indicating a moderating effect. Simple slope analyses were conducted to further interpret this effect. As shown in Figure 2, the positive association between social media use and perceived social support was weaker in the low education level group (−1 *SD, B* = 0.144, *p* < 0.001) and stronger in the high education level group (+1 *SD, B* = 0.328, *p* < 0.001). These findings indicate that higher education level strengthened the positive association between social media use and perceived social support, supporting Hypothesis 5.

## 4 Discussion

This study examined the relationship between social media use and depression among middle-aged and older adults in China. It also explored the potential mechanisms underlying this relationship based on social cognitive theory. The results indicated a negative association between social media use and depression in this population. Health literacy and perceived social support mediated this association. In addition, education level moderated the links between social media use and both health literacy and perceived social support.

### 4.1 Relationship between social media use and depression

This study found a significant negative association between social media use and depression among middle-aged and older adults in China. This finding is consistent with prior research (2, 13) and suggests a potential positive effect of social media in this population. Previous studies on social media use and mental health have mainly focused on younger users and emphasized possible adverse effects (8, 9). By focusing on middle-aged and older adults, the present study helps to expand the population coverage of existing research. Middle-aged and older adults differ from younger users in both patterns and motivations of social media use (13). They are more likely to use social media to maintain social ties and to obtain practical information (26, 27), rather than for identity construction or social comparison. Such use orientations may reduce negative emotions related to comparison pressure and self-evaluation. Social media use may also benefit the mental health of middle-aged and older adults by promoting cognitive engagement and emotional regulation (68, 69). Engaging with news, health knowledge, and daily-life information provides continuous cognitive stimulation and helps maintain information-processing capacity. In addition, entertainment content and communication opportunities can help improve mood and promote social participation, which may reduce the risk of depression (70). China's unique media regulation and information environment may further shape these effects (61). Under the country's strict content management and information censorship (71), middle-aged and older users are exposed to relatively stable information. This context may reduce anxiety caused by information overload or misinformation. Notably, this study employed the Social Media Use Integration Scale (60), which allows for a more accurate assessment of the extent to which individuals integrate social media into their daily lives. In contrast, many previous studies relied on single frequency indicators or fragmented activity items (2, 13), which may underestimate the overall role of social media. Taken together, these findings highlight the importance of population heterogeneity in examining the association between social media use and mental health. Age groups differ in use motives, social needs, and information exposure. Distinguishing younger adults from middle-aged and older adults may allow a more accurate assessment of psychological effects and provide more targeted implications for public health interventions.

### 4.2 Roles of health literacy and perceived social support

This study found that health literacy and perceived social support mediated the association between social media use and depression, while education level played a moderating role. Specifically, social media use was associated with higher health literacy and greater perceived social support, which in turn were related to lower levels of depression. At the same time, education level strengthened the associations of social media use with health literacy and perceived social support. Previous studies reported a negative association between social media use and depression among middle-aged and older adults in China (2, 13), yet the underlying mechanisms remain unexplored. Drawing on social cognitive theory, the present study provides a more explanatory framework. It posits that behavior, personal factors, and environmental influences interact reciprocally, potentially shaping health outcomes (15, 33). From a cognitive perspective, social media provides greater access to health information (36). Through observational learning, individuals improve their ability to understand and apply health knowledge, thereby enhancing health literacy. For example, middle-aged and older adults may learn chronic disease management or access medical service information through short-video platforms or online learning groups (72, 73). From a social perspective, social media offers new opportunities for social interaction (28). It enables users to participate in interest-based groups and facilitates emotional and informational support. Such interactions, including online greetings, interactive feedback, and group discussions, may help alleviate loneliness and feelings of helplessness (74, 75). The moderating role of education level further highlights the importance of personal factors in social cognitive theory. Middle-aged and older adults with higher education tend to have stronger abilities in information comprehension and evaluation. They are more likely to transform online resources into usable health knowledge and to establish stable online relationships. As a result, they may gain greater health benefits from social media use (51). These findings not only advance the theoretical understanding of the relationship between social media use and mental health but also provide implications for intervention practices and public policy. Digital skills and information comprehension among middle-aged and older adults should be strengthened to improve the effectiveness of online health information use (76). Online interactions centered on family, community, and peer networks should be encouraged to enhance social connection and emotional exchange (31, 77). From a policy perspective, differences in accessibility and adaptability across education groups should be considered in digital health and aging services. Stratified design and targeted interventions may help social media better serve mental health promotion among middle-aged and older adults (51).

### 4.3 Limitations

While providing evidence on the mechanisms linking social media use and depression among middle-aged and older adults in China, this study has several limitations. First, the data are cross-sectional, which prevents determination of causal or bidirectional relationships between social media use and depression The association may be reciprocal, with lower depression potentially leading to higher social media use. Future research should adopt longitudinal or experimental designs to clarify temporal order and test potential causal and bidirectional mechanisms. Second, all key variables were measured via self-report. These measures may be subject to recall bias and social desirability effects. Future research could incorporate multi-source data or objective indicators, such as platform usage records or informant reports, to improve measurement accuracy. Third, although several key sociodemographic variables were controlled, other potential confounders, such as physical health status and cognitive function, were not included due to data limitations. Therefore, residual confounding cannot be entirely ruled out. Future studies should include more comprehensive covariates to further reduce this risk. Fourth, the sample was drawn from a province in China, so caution is needed when applying these findings to other regions. In particular, as an island province Hainan is one of the provinces with the best air quality in China (78). Air quality has been shown to be associated with depression among older adults (79), and such regional environmental differences may influence the observed relationships. To further strengthen the generalizability of the results, future research using nationally representative samples is warranted.

### 4.4 Implications

Despite these limitations, this study makes both theoretical and practical contributions. Theoretically, it develops a model grounded in social cognitive theory to explain the association between social media use and depression. Social media use, health literacy, and perceived social support were integrated into a single analytical framework, highlighting the roles of behavioral factors, cognitive factors, and the social environment in shaping mental health. Specifically, social media use may influence mental health through enhanced health literacy and increased perceived social support. The findings support the applicability of social cognitive theory to digital media contexts and extend it to the Chinese setting, providing new theoretical evidence for the mechanisms linking social media use to mental health. Moreover, as one of the few studies examining these mechanisms among middle-aged and older adults in China, this research is exploratory and provides valuable insights for future studies.

Practically, the findings inform mental health interventions and public health policy for middle-aged and older adults. On the one hand, appropriate use of social media may enhance health literacy and perceived social support, thereby reducing the risk of depression. Digital platforms can thus be considered potential tools for mental health promotion. On the other hand, the moderating role of education level indicates unequal benefits across groups. Interventions should account for differences in educational background and digital competence. For individuals with lower education levels, efforts should focus on improving digital literacy and access to reliable health information. Such strategies may support more precise and equitable mental health promotion.

## 5 Conclusion

This study found a significant negative association between social media use and depression among middle-aged and older adults in China. Health literacy and perceived social support mediated this association. In addition, this association varied by education level. This study not only enriches the empirical evidence on social media and mental health but also provides practical implications for mental health promotion among middle-aged and older adults.

## Data Availability

The original contributions presented in the study are included in the article/supplementary material, further inquiries can be directed to the corresponding author.
